# Amantadine as a Potential Treatment for Marchiafava–Bignami Disease: Case Reports and a Possible Mechanism

**DOI:** 10.1155/2022/4585206

**Published:** 2022-04-11

**Authors:** Leenil Noel, Martin Myers, Tigran Kesayan

**Affiliations:** ^1^University of South Florida Health Morsani College of Medicine, Tampa, USA; ^2^Department of Neurology, University of South Florida Health Morsani College of Medicine, Tampa, FL, USA; ^3^Department of Neurology, James A. Haley Veteran's Affairs Hospital, Tampa, FL, USA; ^4^Department of Neurology, Vanderbilt University Medical Center, 1301 Medical Center Drive, Nashville, TN, USA; ^5^Department of Anesthesiology, Vanderbilt University Medical Center, 1301 Medical Center Drive, Nashville, TN, USA

## Abstract

**Introduction:**

Several reports have described the use of amantadine for managing symptoms in Marchiafava–Bignami disease (MBD); however, amantadine's role for the treatment of MBD symptoms is unclear. Here, we describe 2 patients with MBD who were treated with amantadine and hypothesize a potential mechanism responsible for clinical benefit. *Case 1*. A 38-year-old woman with excessive wine drinking presented with agitation, impaired speech, and a minimally conscious state. MRI revealed lesions in the splenium and genu. After being diagnosed with MBD, she was treated with intravenous thiamine, multivitamins, and 100 mg of amantadine twice a day for 2 weeks. She recovered to near baseline after 3 weeks. *Case 2*. A 54-year-old woman with years of heavy alcohol use presented with sudden bradyphrenia, acalculia, disinhibited behavior, weakness, and urinary incontinence. MRI revealed a large anterior callosal lesion. Two years after initial recovery from MBD, she noted that consuming “energy drinks” resulted in a transient, near-complete resolution of her residual behavioral, fatigue, and language symptoms. 100 mg of amantadine twice a day was trialled. After noted improvement, a further escalation to 200 mgs 3 times a day resulted in significant improvement in language and behavioral symptoms.

**Conclusion:**

Amantadine in addition to vitamins may be beneficial in the treatment of MBD. It is possible that the dopaminergic effect of amantadine leads to improved recovery and function in dopamine-mediated pathways, including mesocortical and mesolimbic pathways during initial recovery, as well as improved speech, behavior, and fatigue in the following months. The role of amantadine in the treatment of MBD warrants further study.

## 1. Introduction

Marchiafava–Bignami disease (MBD) is described as demyelination and necrosis of the corpus callosum seen among individuals with chronic alcohol abuse and among those with other conditions, such as diabetes or malnutrition [1–4].

The diagnosis of MBD is clinical, in addition to characteristic brain MRI findings. While a low thiamine level can be helpful for making the diagnosis, there is no established laboratory value associated with MBD. Patients typically present with altered mentation or unconsciousness, impaired speech, gait, motor function, and rigidity [2, 3, 5]. Clinical presentation ranges from status epilepticus to disconnection syndromes, such as unilateral apraxic agraphia or hemispatial neglect [2, 6, 7]. MRI often reveals T1-weighted sequence hypointensities and T2-weighted and fluid-attenuated inversion recovery (FLAIR) sequence hyperintensities in the corpus callosum [8]. During acute presentation, there is often restricted diffusion within the corpus callosum [9].

There are no established guidelines for the treatment of MBD. The therapeutic approach involves supportive care, vitamin and nutrient replacement, and the treatment of sequelae, such as seizures [5, 6, 8]. In the literature, thiamine replacement is the most supported treatment. In a review of 144 patients with MBD, those treated with thiamine had significantly better outcomes than those who were not [5]. Intravenous steroids have also been used, but their role in treating MBD is unclear [1, 5].

Two case reports have described the use of amantadine for the management of MBD [10, 11]. Amantadine is an organic compound that modulates dopamine receptor activity and dopamine release, inhibits dopamine reuptake, and has dopamine agonist and N-methyl-D-aspartate (NMDA) antagonist properties [12–15]. These properties may play a role in the use of amantadine for the treatment of fatigue from multiple sclerosis as well as its potential for facilitating cognitive recovery and function after a traumatic brain injury [16, 17]. The role of amantadine for treating MBD is unclear, and there are neither known nor hypothesized mechanisms as to why this medication may provide a therapeutic benefit.

Here, we present two patients with MBD who have been treated with amantadine and discuss a possible mechanism for amantadine's potential role in the treatment of MBD.

## 2. Case Presentation

Both patients provided informed consent for their cases to be described.

### 2.1. Case 1

**Figure 1:**
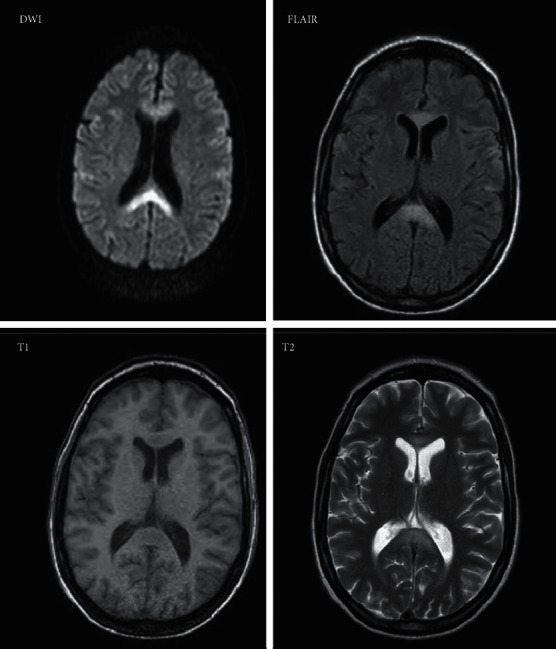
An MRI of the brain with a diffusion-weighted imaging (DWI) sequence showing restricted diffusion in the genu and splenium of the corpus callosum. A T1-weighted sequence shows hypointensity in the genu and splenium with corresponding hyperintensity on T2-weighted and T2-weighted fluid-attenuated inversion recovery (T2-FLAIR) images T2 and FLAIR. There is no enhancement noted with gadolinium (not shown).

A 38-year-old woman presented with agitation, impaired speech, and a minimally conscious state. She had a history of declining cognition for several months and impaired speech and ambulation for three weeks prior to presentation. She had over a year of excessive wine drinking and poor diet. MRI, shown in Figure 1, obtained the day after her presentation revealed restricted diffusion in the corpus callosum, most prominent in the splenium and genu; there were FLAIR sequence hyperintensities and T1-weighted sequence hypointensity in the same regions. Cerebrospinal fluid examination was unremarkable. Serum sodium and serum glucose were both within the normal limits of the testing laboratory, between 135–145 mml/L and 3.9–5/6 mmol/L, respectively. Serum thiamine was less than 7 nmol/L (normal 8–30 nmol/L). A diagnosis of MBD was made, and she was treated with intravenous thiamine, multivitamins, and three days of intravenous methylprednisolone during the first week of hospitalization. During the second week, intravenous thiamine and multivitamins were continued, and she began taking 100 mg of amantadine orally twice a day for a two-week course. Three weeks after her presentation, she recovered her speech, returned to near-baseline cognitive status, and regained mobility. She continued to have slight left hemispatial neglect found on a line bisection test. One year later, she continued to have left apraxic agraphia. Her case is discussed in more detail by Kesayan and colleagues elsewhere [6, 7].

### 2.2. Case 2

**Figure 2:**
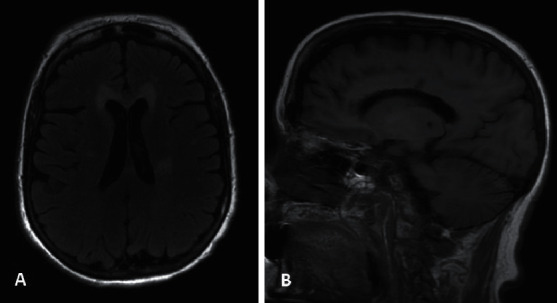
(a) An axial fluid-attenuated inversion recovery (FLAIR) sequence demonstrating a hyperintense lesion in the genu of the corpus callosum and smaller lesions, most notably in the left corona radiata area. (b) A sagittal T1-weighted image demonstrating a hypointense lesion in the genu and body of the corpus callosum.

A 54-year-old woman presented with sudden onset of the right arm, leg, and face weakness, bradyphrenia, difficulty with calculation, urinary incontinence, child-like behavior, and significant fatigue. She was diagnosed with stroke and discharged from the hospital. Her weakness improved with physical therapy. In the several years preceding her stroke diagnosis, she reported heavy alcohol consumption. Two years after her initial symptoms, she presented to our clinic with bradyphrenia, acalculia, and behavioral changes. MRI, shown in Figure 2, obtained at the time of her presentation at our clinic revealed a large anterior corpus callosum lesion along with left greater than right corona radiata white matter lesions. Serum sodium and glucose levels were within normal limits during her initial presentation, where a diagnosis of stroke was made, and upon presentation to our clinic. Thiamine levels were not available from her initial presentation. Given her presentation along with her MRI findings, she was diagnosed with MBD.

She and her family explained that consumption of “energy drinks” resulted in temporary near-complete resolution of her behavior, fatigue, and language symptoms immediately after consumption. Given amantadine's use for fatigue in multiple sclerosis as well as previous reports of its use for MBD, it was hypothesized that amantadine could be beneficial and thus was trialled at 100 mg twice daily. There was some reported improvement in her fatigue and cognitive changes, so a further escalation to 200 mg of amantadine thrice daily was trialled. This resulted in significant improvements in language and behavior symptoms, but ultimately, auditory and visual hallucinations from amantadine required discontinuation of the medication. Considerations of further dose adjustments or other therapies were made, but the patient was lost to follow up before these could be trialled.

## 3. Discussion

We present the third and fourth reported patients with MBD treated with amantadine. Clinical improvement was noted in both of our patients, although they were treated with amantadine at different time courses from their initial symptoms.

Two other case reports have described the use of amantadine to treat MBD. In 1998, Gass et al. described a 43-year-old patient treated with a five-day course of intravenous amantadine who had significant improvement in mental status and some motor function improvement. In 2006, Staszewski et al. described a 52-year-old patient treated with 400 mg/day of intravenous amantadine who experienced improvement in cognitive and motor function.

The pathophysiology of MBD is not clearly understood. Demyelination due to nutrient deficiency, alcohol, or hyperosmolar states have been hypothesized [4, 8, 18]. The role of amantadine within the hypothesized potential mechanisms is unclear, and the mechanism by which amantadine may have some effect in symptoms seen with MBD could be unrelated to demyelination and its potentials causes. Furthermore, the exact contribution of amantadine in these cases cannot be truly estimated due to confounding medications and the elaborate timeline of each patient's treatment course.

Amantadine is used for traumatic brain injuries and is a commonly prescribed medication for patients with impaired consciousness who are undergoing neurorehabilitation [17, 19]. Amantadine has been shown to improve the speed of recovery of function after traumatic brain injury-related impairments of consciousness, and this benefit was seen across a wide range of impairment severities [17]. In the immediate phases after insult, the brain undergoes a short period of neuronal excitability, followed by a longer period of hypoexcitability, which includes depletion of dopamine along with other neurotransmitters [17]. Amantadine has been shown to modulate dopamine receptors, leading to dopamine release, and act to inhibit dopamine reuptake [12–15]. Furthermore, positron emission tomography studies have shown that amantadine leads to an increase in dopamine receptor availability in the striatum as well as increased prefrontal cortical metabolic activity [20, 21]. As such, it is hypothesized that the neurological benefits of amantadine may be due to its role in improving enhanced neurotransmission in the dopamine-dependent circuits governing arousal, drive, and attentional functions. It is possible that a similar mechanism may play a role in clinical improvement in MBD.

Our first patient was treated with thiamine and multivitamins, in addition to amantadine, along with a 3-day course of steroids. As such, it is not known to what degree amantadine played a role in her significant improvement from a stuporous state to near-complete recovery over 3 weeks. Of note, in the 2 previous cases of patients with MBD who were treated with amantadine, as well as the case of the patient treated with memantine, patients were also treated with B vitamins, with 2 of those 3 patients also being treated with piracetam [10, 11].

Our second patient experienced improved cognition and behavior after consuming “energy drinks.” Although the exact ingredients of the drinks were not known or able to be verified by the patient or her family, it was presumed that some form of the stimulant effect from caffeine benefited the patient. It is possible that dopaminergic effects are responsible for this improvement in behavior. Dysfunction of the dopaminergic mesocortical and mesolimbic pathways were hypothesized for this patient in part because of her symptoms and in part because of the predominant anterior corpus callosal lesions seen on MRI. It is possible that the dopaminergic effects of amantadine contributed to improved recovery and function in dopamine-mediated pathways further leading to improved recovery during the acute phase, as well as improved speech, behavior, and fatigue months after initial recovery. Support for this theory comes from studies showing increased arousal and cognition, as well as increased prefrontal cortical metabolic activity and striatal D2 dopamine receptor availability, after amantadine use [15, 20, 21].

The patient in our case 2 had a left greater than right corona radiata lesion in addition to a lesion in her corpus callosum; these lesions likely explain her hemibody weakness. MRI findings in MBD classically include corpus callosum lesions, but extracallosal lesions within the white matter of any lobe, in addition to callosal lesions, can also be present among a small percentage of patients [5, 8, 10]. Furthermore, unilateral motor and praxis symptoms have also been described from callosal lesions [6, 22].

Amantadine, with its dopamine agonist and NMDA receptor antagonist properties, may be a potential therapeutic agent for the treatment of MBD for improved clinical outcomes in the acute or chronic phases of disease. While we cannot currently reliably conclude amantadine's usefulness in these cases of MBD, the improvements observed in these and other reported patients with MBD treated with amantadine certainly warrants further studies to better characterize amantadine's role as a treatment option.
